# Glutathione S-transferase M1 and T1 genetic polymorphisms, alcohol consumption and breast cancer risk

**DOI:** 10.1038/sj.bjc.6600708

**Published:** 2003-01-28

**Authors:** T Zheng, T R Holford, S H Zahm, P H Owens, P Boyle, Y Zhang, B Zhang, J P Wise, L P Stephenson, F Ali-Osman

**Affiliations:** 1 Department of Epidemiology and Public Health, Yale University School of Medicine, 60 College, New Haven, CT, USA; 2 Division of Cancer Epidemiology and Genetics, National Cancer Institute, Rockville, MD 20892, USA; 3 Department of Epidemiology and Biostatistics, European Institute of Oncology, Milan 20141, Italy; 4 Department of Epidemiology and Biostatistics, McGill University, Montreal, Canada H3A1A2; 5 Department of Neurosurgery, University of Texas at MD Anderson Cancer Center, Houston, TX, USA

**Keywords:** alcohol drinking, breast cancer, case–control, GST genotypes

## Abstract

Alcohol consumption has been inconsistently associated with breast cancer risk. Recent studies suggest that genetic polymorphisms of glutathione *S*-transferases (GSTs) may modify this relation. To determine if breast cancer risk is associated with GSTM1 and GSTT1 genetic polymorphisms, and to evaluate the effect modification between GST genotypes and alcohol consumption in the risk of breast cancer, we conducted a case–control study in the state of Connecticut in the period 1998 and 2001. Cases were histologically confirmed, incident breast cancer patients in New Haven County, CT. Controls were randomly selected from women histologically confirmed to be without breast cancer. The study results show that, while GSTM1 genotypes were not associated with breast cancer risk, GSTT1-null genotype was associated with a significant 90% increased risk for postmenopausal women (OR=1.9, 95% CI 1.2–3.0). Analysis by GST genotypes and alcohol consumption shows that GSTM1A ever-drinking women had a 2.5-fold (OR=2.5, 95% CI 1.1–5.5) increased risk of breast cancer compared to the GSTM1A never-drinkers, and the risk increases with duration and daily amount of alcohol consumption. Postmenopausal women with GSTT1-null genotype, who consumed a lifetime of >250 kg of spirit-equivalents, had an almost seven-fold increased risk (OR=6.8, 95% CI 1.4–33.9), and drinking commencing at younger ages appears to carry a higher risk. An OR of 8.2 (95% CI 1.2–57.4) was observed for those with GSTM1A, and GSTT1-null genotypes who had consumed a lifetime of >250 kg of spirit-equivalents. In conclusion, alcohol consumption may increase breast cancer risk among those who carry susceptible GST genotypes.

Human glutathione *S*-transferases (GSTs) are considered to be particularly important for detoxifying many carcinogenic compounds and reactive intermediates that may be breast carcinogens (Ketterer, 1988; Hayes and Pulford, 1995; Smith *et al*, 1995; Rebbeck, 1997). Subjects with different GST genotypes may therefore have different susceptibilities to environmental exposures.

About 50% of the Caucasian population carries a homozygous deletion of the GSTM1 locus, resulting in an inactive gene product (null genotype), and the lack of functional GSTM1 enzyme activity. Studies have shown that individuals who inherit the GSTM1 null genotype are not capable of conjugating and detoxifying specific substrate epoxide intermediates (Wiencke *et al*, 1990). Thus, the absence of the GSTM1 gene should increase cancer risk from environmental exposure while the presence of the intact GSTM1 gene would be protective for cytogenetic damage and carcinogen-derived DNA adduct formation. The GSTT1 gene, located on chromosome 6, is absent from about 40% of the population (Pemble *et al*, 1994). GSTT1 has also been involved in the glutathione-dependent detoxification. Similar to GSTM1, GSTT1 has significant activity towards epoxides, suggesting that individuals without both GSTM1 and GSTT1 may be at a particularly high risk of cancer (Wiencke *et al*, 1995). Moreover, the results of recent studies suggest that for alcohol drinkers interactions with GSTM1 and GSTT1 deletion polymorphisms may play an important role in individual susceptibility to breast cancer (Helzlsouser *et al*, 1998; Park *et al*, 2000).

In this case–control study, we sought to determine if the polymorphisms of GSTM1 and GSTT1 modify the relationship between alcohol drinking and breast cancer risk based on detailed information on lifetime alcohol consumption.

## Materials and methods

### Study subjects

This study used blood samples and data collected from a recently completed case–control study of female breast cancer in Connecticut. The detailed description of the study population and the methods have been described elsewhere (Zheng *et al*, 2000,^2002^). Briefly, cases were histologically confirmed, incident breast cancer patients who had a breast-related surgery at the Yale-New Haven Hospital (YNHH), in New Haven County, Connecticut in 1994–1997. Patients were 30–80 years old. Potentially eligible cases were identified using computerised patient information from YNHH, where records of all newly completed breast-related surgeries are kept. We consecutively selected all breast cancer patients who met the study eligibility requirements as described above. A total of 326 incident breast cancer patients were recruited. The participation rate was 77% for patients.

In order to avoid selection of controls with nondiagnosed early-stage breast cancer or precancerous conditions, we randomly selected controls from the same computerised files from those who had had breast-related surgery but were histologically confirmed to be without breast cancer. A total of 347 controls were selected and frequency matched to the cases by age, within 5-year intervals (30–34,…). The participation rate was 71% for controls.

### Interviews

After approval by each subject's hospital and physician, potential participants were approached by letter and then by phone. Those who consented were interviewed by a trained interviewer, either at home or at a location convenient for the patient. A standardised, structured questionnaire was used to obtain information on alcohol drinking, tobacco smoking, menstrual and reproductive history, lactation history, past medical history, family cancer history, occupation, diet and demographic factors. Information on lifetime alcohol consumption included the type of alcoholic beverage used, age at which drinking commenced, amount of alcohol consumed per day, the frequency with which the subject consumed each type of alcoholic beverage, the duration during which each type of alcoholic beverage was consumed and total duration of drinking.

### Blood collection and laboratory analysis of GST genotypes

As recently reported elsewhere (Zheng *et al*, 2002), blood clot samples were sent in batches to the study laboratory at the University of Texas at MD Anderson Cancer Center to isolate high molecular weight genomic DNA for GST genotyping. DNA purity and yield were assessed by determining the optical densities at 260 and 280 nm. Genotyping of GSTM1 and GSTT1 was performed using a combination of PCR and RFLP analysis, using a modification of a previously described method (Fryer *et al*, 1993a,1993b). Amplification was performed with the GSTM1- and GSTT1-specific primers. The PCR product was electrophoresed in 2% agarose, stained in 0.5% ethidium bromide and photographed under UV illumination. Cell lines (human malignant gliomas and breast carcinoma) available in our study laboratory and representing GSTT1-positive, GSTT1-null, GSTM1A, GSTM1B and GSTM1-null polymorphisms were used as positive controls. Quality control procedures implemented for the GST genotype analyses included the running of controls of stable human cancer cell lines with a known polymorphic GST gene and reanalysing samples that yielded ambiguous results. Samples were coded and batched at Yale, and the laboratory personnel at the MD Anderson Cancer Center were blinded to their identity.

### Data analysis

Unconditional logistic regression was used to estimate the association between GSTM1 and GSTT1 genetic polymorphisms and breast cancer risk, to evaluate the putative modification by GST genotypes of the effect of alcohol drinking on the risk of breast cancer, and to control for potential confounders. The average adult lifetime daily consumption of alcohol in grams and the total lifetime consumption in kilograms of spirit- equivalents were calculated by taking into account the frequency, the amount and the duration of consumption of each type of alcoholic beverage. For these analyses, grams of beer and fruit wine were converted into grams of spirit-equivalents as follows: grams of beer were divided by 8, grams of wine by 2, according to the approximate ratio of alcohol contents in beverages. Each amount was then multiplied by the weekly reported frequency of consumption of each type of alcoholic beverage and further divided by 7 to estimate average daily consumption. Finally, the total lifetime kilograms of spirit-equivalent consumption was estimated.

Variables included in the final model were age (as a continuous variable), body mass index (<21, 21–24, ≥25 weight in kilograms/square of height in meters), lifetime months of lactation (0, 1–5, 6–11, >11 months), age at first full-term pregnancy (nulliparous, <20, 20–25, >25 years), family breast cancer history and menopausal status. Additional adjustment for other variables such as age at menarche, age at menopause, number of live births and use of exogenous hormones did not result in material change for the observed association; therefore, these variables were not included in the final model. Maximum likelihood estimates of the parameters were obtained using SAS (SAS Institute, 1990).

## Results

Neither of the GSTM1 allelotypes were significantly associated with breast cancer risk for all women combined or among pre- or postmenopausal women (data not shown). Among women with GSTT1-null genotype, however, there was a significant, 50%, increase in breast cancer risk for all women combined (OR=1.5, 95% CI 1.0–2.2). Further analysis by menopausal status indicates that this risk was limited to postmenopausal women (OR=1.9, 95% CI 1.2–3.0).

Table 1

**Table 1:** GSTM1 genotypes, alcohol consumption and breast cancer risk

	**GSTM1-A**		**GSTM1-B**		**GSTM1-null**	
**Alcohol drinking**	**Ca/Co^a^**	**OR^b^ (95% CI)**	**Ca/Co^a^**	**OR^b^ (95% CI) **	**Ca/Co^a^**	**OR^b^ (95% CI)**
Never	13/23	1.0	5/4	1.0	20/23	1.0
Ever	93/79	**2.5 (1.1/5.5)^c^**	36/40	0.8 (0.2/3.3)	145/150	1.1 (0.6/2.3)
						
Age when started drinking (y)						
<18	18/19	2.1 (0.8/5.6)	5/11	0.4 (0.1/2.2)	26/28	1.4 (0.6/3.3)
18–24	56/47	**2.4 (1.0/5.6)**	25/24	0.9 (0.2/3.9)	97/96	1.2 (0.6/2.4)
>24	19/13	**3.2 (1.1/9.1)**	6/5	0.9 (0.1/6.1)	22/26	0.9 (0.4/2.1)
*P* for trend		0.14		0.86		0.99
						
Spirit-equivalent drinking per day (g)						
<80	16/19	2.1 (0.8/6.0)	9/11	0.6 (0.1/3.1)	35/31	1.2 (0.5/2.7)
80–150	32/29	2.0 (0.8/5.1)	11/16	0.6 (0.1/3.0)	54/67	1.0 (0.5/2.0)
>150	45/31	**3.1 (1.3/7.6)**	16/13	1.1 (0.2/5.3)	56/52	1.3 (0.6/2.8)
*P* for trend		0.07		0.15		0.87
						
Years of drinking						
<15	13/19	1.7 (0.6/4.7)	9/5	1.9 (0.3/13.3)	23/23	1.3 (0.5/3.1)
15–30	38/41	1.9 (0.8/4.5)	14/22	0.6 (0.1/3.0)	62/74	1.2 (0.6/2.6)
>30	42/19	**4.2 (1.6/10.9)**	13/13	0.8 (0.2/3.8)	60/53	1.0 (0.5/2.2)
*P* for trend		0.01		0.31		0.93
						
Lifetime kilograms of spirit-equivalent drinking						
<70	20/24	1.9 (0.7/5.0)	10/14	0.5 (0.1/2.8)	37/44	1.0 (0.4/2.1)
70–250	23/28	1.7 (0.6/4.4)	10/11	0.9 (0.2/4.8)	44/34	1.6 (0.7/3.6)
>250	50/27	**3.4 (1.4/8.2)**	16/15	0.9 (0.2/4.1)	64/72	1.0 (0.5/2.1)
*P* for trend		0.03		0.19		0.87

a Cases/controls.

b Adjusted for age, BMI (<21, 21–24, >24), age at first full-term pregnancy nulliparous (<20, 20–25, >25 y), lifetime duration of lactation (never, 1–5, 6–11, >11 months), family breast cancer history and menopausal status.

c Bold entries for statistical significance.

presents the association between alcohol consumption and breast cancer risk by GSTM1 genotype. There was no increased risk associated with alcohol consumption for women with GSTM1B and GSTM1-null genotypes. There was, however, a significantly increased risk for women with GSTM1A genotypes. As shown in Table1, GSTM1A ever-drinking women had a 2.5-fold (OR=2.5, 95% CI 1.1–5.5) increased risk of breast cancer compared to the GSTM1A never-drinkers. Furthermore, the risk seems to increase with duration and daily amount of alcohol consumption. For those who drank more than 150 g of spirit-equivalents daily, the OR was 3.1 (95% CI 1.3–7.6). For those who had more than 30 years of alcohol consumption, the OR was 4.2 (95% CI 1.6–10.9). For those with a lifetime of more than 250 kg of spirit-equivalent consumption, the OR was 3.4 (95% CI 1.4–8.2). Further stratification by menopausal status indicates that the observed significant association is limited to postmenopausal women (data not shown). For example, ever-drinking postmenopausal women, who had a GSTM1A genotype, had a three-fold (OR=3.1, 95% CI 1.1–8.4) significantly increased risk of breast cancer, while ever-drinking premenopausal women did not (OR=1.4, 95% CI 0.3–6.1).

The relation between alcohol consumption and breast cancer risk by GSTT1 genotype is presented in Table 2

**Table 2:** GSTT1 genotypes, alcohol consumption and breast cancer risk

	**GSTT1-positive**		**GSTT1-null**	
**Alcohol drinking**	**Ca/Co^a^**	**OR^b^ (95% CI)**	**Ca/Co^a^**	**OR^b^ (95% CI)**
Never	30/41	1.0	10/11	1.0
Ever	192/219	1.2 (0.7/2.0)	85/63	1.6 (0.6/4.4)
				
Age when started drinking (y)				
<18	37/50	1.2 (0.6/2.2)	14/9	2.1 (0.5/8.2)
18−24	126/132	1.3 (0.7/2.3)	52/41	1.5 (0.5/4.5)
>24	29/37	1.0 (0.5/2.0)	19/13	1.5 (0.5/5.1)
*P* for trend		0.92		0.91
				
Spirit-equivalent drinking per day (g)				
<80	41/51	1.0 (0.5/1.9)	19/13	1.8 (0.5/6.1)
80–150	62/92	0.9 (0.5/1.7)	36/26	1.5 (0.5/4.4)
>150	89/76	1.6 (0.9/2.9)	30/24	1.6 (0.5/5.1)
*P* for trend		0.20		0.46
				
Years of drinking				
<15	32/32	1.4 (0.7/2.9)	13/17	1.1 (0.3/3.7)
15–30	74/114	0.9 (0.5/1.7)	42/29	2.1 (0.7/6.6)
>30	86/73	1.4 (0.8/2.6)	30/17	1.5 (0.5/4.8)
*P* for trend		0.49		0.38
				
Lifetime kilograms of spirit-equivalent drinking				
<70	45/62	1.0 (0.5/1.9)	22/24	1.1 (0.3/3.4)
70–250	51/54	1.4 (0.7/2.6)	27/20	1.4 (0.4/4.5)
>250	96/103	1.2 (0.7/2.2)	36/19	2.4 (0.8/7.6)
*P* for trend		0.20		0.04

See Table 1.

. There was no increased risk associated with alcohol consumption for women with GSTT1-positive genotype; however, there was a trend of an effect for women with GSTT1-null genotype, as evidenced (Table2) by a 60%, albeit statistically insignificant, increase in breast cancer risk (all subjects combined) in women who had consumed alcohol (‘ever-drinkers’). Stratifying women by menopausal status and alcohol consumption revealed that this apparent effect was significant for postmenopausal women.

The data suggest that the more alcohol that postmenopausal women with GSTT1-null genotype consume, the greater their risk of breast cancer (Table 3

**Table 3:** Alcohol drinking and breast cancer risk for subjects with GSTT1-null genotype

	**Premenopausal**		**Postmenopausal**	
**Alcohol drinking**	**Ca/Co^a^**	**OR^b^ (95% CI)**	**Ca/Co^a^**	**OR^b^ (95% CI)**
Never	3/4	1.0	7/7	1.0
Ever	21/30	0.8 (0.1/4.9)	64/33	2.3 (0.6/8.3)
				
Age when started drinking (y)				
<18	5/6	1.0 (0.1/9.9)	9/3	3.2 (0.5/21.9)
18–24	12/19	0.8 (0.1/5.0)	40/22	2.4 (0.6/9.6)
>24	4/5	0.9 (0.1/8.0)	15/8	1.8 (0.4/8.3)
*P* for trend		0.82		0.99
				
Spirit-equivalent drinking per day (g)				
<80	7/6	1.4 (0.2/11.7)	12/7	1.9 (0.4/9.4)
80–150	6/10	0.8 (0.1/6.1)	30/16	2.2 (0.6/8.6)
>150	8/14	0.6 (0.1/4.1)	22/10	3.1 (0.6/15.1)
*P* for trend		0.64		0.18
				
Years of drinking				
<15	6/8	1.2 (0.1/9.0)	7/9	0.9 (0.2/5.0)
15–30	15/18	1.2 (0.2/7.8)	27/11	2.9 (0.6/13.1)
>30	0/4		30/13	2.6 (0.6/10.8)
*P* for trend		0.25		0.11
				
Lifetime kilograms of spirit-equivalent drinking				
<70	8/11	0.9 (0.1/5.7)	14/13	1.0 (0.2/4.7)
70–250	8/8	1.1 (0.1/8.3)	19/12	1.5 (0.3/7.3)
>250	5/11	0.6 (0.1/4.6)	31/8	6.8 (1.4/33.9)
*P* for trend		0.64		0.03

a Cases/controls.

b Adjusted for age, BMI (<21, 21–24, >24), age at first full-term pregnancy nulliparous (<20, 20–25, >25 y), lifetime duration of lactation (never, 1–5, 6–11, >11 months), family breast cancer history.

). Specifically, Table3 shows that women who consumed a lifetime of more than 250 kg of spirit-equivalents had an almost seven-fold increased risk of breast cancer (OR=6.8, 95% CI 1.4–33.9). This was supported by observations that the risk increases with increasing duration and daily amount of alcohol consumption, with drinking commencing at younger ages appearing to carry a higher risk, although these results were not statistically significant.

Table 4

**Table 4:** GSTM1, GSTT1 genotypes, alcohol consumption and breast cancer risk

	**Lifetime kilograms of spirit-equivalent drinking **			
	**⩽250**		**>250**	
**GST genotypes**	**Ca/Co^a^**	**OR**	**Ca/Co^a^**	**OR^b^ (95% CI)**
GSTT1-Null				
GSTM1-Null	27/26	1.0	19/14	1.3 (0.4/4.0)
GSTM1-A	23/20	1.0	11/2	**8.2 (1.2/57.4)**
GSTM1-B	7/7	1.0	6/2	8.9 (0.6/130.7)
				
GSTT1-Positive				
GSTM1-Null	74/75	1.0	45/58	0.8 (0.5/1.3)
GSTM1-A	33/55	1.0	39/25	**2.3 (1.1/4.7)**
GSTM1-B	18/22	1.0	10/13	0.9 (0.3/2.7)

See Table 1.

presents the joint effects of GSTM1, GSTT1 genotypes and alcohol consumption (lifetime kilograms of spirit-equivalent consumption) in the development of breast cancer. A statistically significant eight-fold (OR=8.2, 95% CI 1.2–57.4) increased risk of breast cancer was observed for those with GSTM1A, and GSTT1-null genotypes who had consumed a lifetime total of more than 250 kg of spirit-equivalents. Subjects with GSTT1-positive and GSTM1A genotypes who consumed a lifetime total of more than 250 kg of spirit-equivalents also showed a significantly increased risk of breast cancer (OR=2.3, 95% CI 1.1–4.7). No significantly increased risk was observed for other GSTM1 and T1 genotype combinations.

## Discussion

The results of this study suggest that there is an increased risk of breast cancer associated with alcohol consumption among those carrying certain genotypes. We found that postmenopausal women with a GSTM1A genotype have an increased risk of breast cancer when they drink, and the risk increases with increasing amount and duration of alcohol consumption. We also found that breast cancer risk is increased for postmenopausal women with the GSTT1-null genotype who consume more than 250 kg of spirit-equivalents. An eight-fold significantly increased risk was observed among heavier drinkers who had GSTM1A and GSTT1-null genotypes.

Alcohol consumption has previously been associated with breast cancer risk, but the results have been inconsistent (Rosenberg *et al*, 1993). It has been postulated that alcohol may influence the risk of breast cancer through effects on pituitary-prolactin secretion, metabolism and clearance of Oestrogen by the liver, or pineal-melatonin production (Willett *et al*, 1989; Hiatt, 1990). Other potential mechanisms may include alcohol-facilitated transport of carcinogens to breast tissue, alcohol disruption of membrane function, or immunoincompetence through the promotion of nutritional deficiencies or liver disease (Rosenberg *et al*, 1993). Alcohol may also induce the formation of free radicals and a rise of lipid peroxidation which can lead to DNA damage (Wright *et al*, 1999).

GSTs are involved in the metabolism of a wide variety of potential carcinogenic compounds, including peroxides, organic epoxides, aromatic amino/nitro compounds and steroids. GST isozymes, such as GSTM1 and GSTT1, have distinct but overlapping substrate specificity as reviewed by Helzlsouser *et al* (1998). Thus, GSTs could interact with alcohol or its contaminants in the development of breast cancer. For example, lack of GST genes could reduce the capacity to conjugate lipid peroxidation products, cytotoxic compounds and free radicals generated during alcohol metabolism (Park *et al*, 2000). Thus, the inconsistent results relating alcohol consumption to breast cancer risk might be explained by genetic differences in detoxification enzymes in the study populations. An increased risk of breast cancer associated with alcohol consumption may be apparent only among those carrying putative high-risk genotypes.

The role of GSTT1 in the risk of breast cancer is supported by earlier studies (Helzlsouser *et al*, 1998; Park *et al*, 2000). Lack of GSTT1-mediated conjugation could result in an increase in the risk for those with exposure to alcohol, its by-products or its contaminants. The biological mechanisms underlying our observations about GSTM1A are less clear. Although previous studies observed a significant interaction between GSTM1-null, GSTT1-null genotypes and alcohol consumption in the risk for breast cancer among premenopausal women (Park *et al*, 2000) and among both pre- and postmenopausal women (Helzlsouser *et al*, 1998), the potential association between individual GSTM1 alleles and breast cancer or their interaction with alcohol consumption in modifying the risk of breast cancer was not investigated. Our findings in this study are, however, consistent with those in several reports indicating potential differences in the effects of GSTM1A and GSTM1B in breast and other cancers (Fryer *et al*, 1993a,1993b; Duncan *et al*, 1995; Inskip *et al*, 1995; Charrier *et al*, 1999). There is also the possibility that GSTM1 may be linked to another gene involved in the metabolism of putative breast carcinogens. Indeed, there are reports indicating that GSTM1 is linked to GSTM3 (Inskip *et al*, 1995) and GSTP1 (Maugard *et al*, 2001), and thus the effect of GSTM1 on cancer susceptibility may be influenced by the expression of GSTP1 and/or GSTM3, suggesting that interactions between GST genes may be a significant factor in determining cancer susceptibility.

A potential limitation of this study and other earlier studies (Zhong *et al*, 1993; Ambrosone *et al*, 1995; Bailey *et al*, 1998; Charrier *et al*, 1999; Garcia-Closas *et al*, 1999) that investigated the association between GST genotypes alone or their interaction with alcohol consumption in the risk of breast cancer is the relatively small sample sizes. Therefore, chance may explain the inconsistent results linking GST genotypes, alcohol drinking and breast cancer. For example, the study by Helzlsouser *et al* (1998) involved 110 cases and 113 controls, and the study by Park *et al* (2000) involved 189 cases and an equal number of controls. Although our study has a sample size of 326 cases and 347 controls, this is still relatively small, especially after stratification by GST genotype and menopausal status. Studies with larger sample size from different populations are clearly needed to address the issue.

In conclusion, this is the first study using detailed information on lifetime alcohol consumption to examine the effect of alcohol consumption on the risk of breast cancer by GST genotypes. The results of this study suggest that alcohol consumption may increase breast cancer risk among those who carry susceptible GST genotypes. A potential effect modification between GST genotypes and alcohol consumption in the development of breast cancer is biologically plausible. If this association is causal, the high frequency of the at-risk GST genotypes in the population means that avoidance of exposure among individuals with these susceptibility genotypes should result in a substantial reduction of breast cancer cases. If future studies demonstrate that alcohol consumption is, indeed, a risk factor for breast cancer among individuals with high-risk GST genotypes, alcohol consumption would be one of the few modifiable risk factors for breast cancer identified to date.
